# Suppurative Inflammation of the Gums and Absorption of the Gums and the Alveolar Process

**Published:** 1899-01

**Authors:** John W. Riggs

**Affiliations:** Hartford, Connecticut.


ARTICLE III.

### SUPPURATIVE INFLAMMATION OF THE GUMS AND ABSORPTION OF THE GUMS AND THE ALVEOLAR PROCESS.

JOHN W. RIGGS, F. A. A. D. S., HARTFORD, CONNECTICUT.

Read before the American Academy of Dental Surgery, New York, October 20th, 1875.

[We take extreme pleasure in giving to our readers the opinion of the late Dr. Riggs upon "Pyorrhœa Alveolaris." Dr. Riggs first instituted a successful treatment of this disease, and the able statement from his own pen of its etiology, pathology and treatment must demand consideration. We hazard the statement that, while volumes have since been written upon the subject, the statements of Dr. Riggs, made twenty three years ago, remain uncontroverted to this day.—*Ed. W. D. J.*]

This disease is called by many the disease of old age, as formerly it was more particularly noticed in persons of advanced years, but at the present day we find the middle-aged and even the young affected by it. Many of the text-books of our speciality consider it hereiditary, or constitutional, or bone disease arising from a scrofulous diathesis. The result, from whatever cause, is most disastrous to the teeth, and in many cases to the health of the patient, for one by one the teeth become loose from loss of bony support, and are plucked out as an intolerable annoyance. If the inflammatory action be great and involve most or all the gum embracing the teeth, pus tinged with blood exudes from around the necks of the teeth on the slightest pressure of the lips or tongue, or in mastication. The oral secretions become vitiated, present a viscid or sanious character, very abundant in quantity during the day, but much more so in the recumbent position of sleep. If the patient reposes on his side, these exudations flow out of the corner of the mouth over the pillow and present in the morning a dried, yellow discoloration, often tinged with blood and covering a space as large as one's hand. If the patient reclines on his back, the diseased mass flows back into the fauces and is unconsciously swallowed, there to work a greater mischief by breaking down the tone of the stomach and poisoning its contents. I apprehend there can be little question or dispute among physicians or physiologists as to the deleterious effect of this putrefactive ferment on the digestive organs. Indeed, of the many intelligent practitioners, the writer has met only one who maintained that these purulent exudations were harmless—yea, absolutely nutritious; that the gastric juice transmuted every noxious characteristic into healthy pabulum of the blood.

Notwithstanding this single opinion, the diagnosis of a number of cases for treatment fully sustains in the former conclusions, confirmed, as they are in every instance, by the rapid recovery of the general health of the patient by restoring the particular health of the mouth.

None but the vigorous constitutions can withstand this type of the disease. The appetite begins to fail; even the odor of cooking the most savory food is offensive. A cup or two of strong coffee and a few crackers constitute the sole breakfast; languor and ennui are substituted for cheerfulness and vigor of mind and body; neuralgic pains sweep up through the face to both hemispheres of the brain and are intensified by a recumbent position, thus banishing sleep. The patient paces his room at night, or reclines as well as he may, until in his agony he cries out, as Macbeth to his physician, "Canst thou not minister to a mind diseased, pluck from the memory a rooted sorrow, raze out the written troubles of the brain, and with some sweet oblivious antidote cleanse the stuffed bosom of that perilous stuff, which weighs upon the heart?"

The disease also aggravates, if it does not cause, nasal catarrh. One of my patients, a physician, the best authority, of course, strenuously maintains that the restoration of his gums, teeth and mouth to health relieved him entirely of a violent catarrh, upon which he had exhausted all known remedial agents. One patient, besides great prostration from aggravated symptoms, became nearly blind. She could not see to read ordinary print, and could only tell the number of windows in a room by the mass of light from each. She could not walk up a flight of steps without assistance. I had the pleasure, some three years ago, of presenting this patient for examination before that very enthusiastic society of workers, the Brooklyn Dental Association. She was then well, sufficiently so to walk several miles daily. Gums and mouth healthy; appetite good, and fast improving in general condition. As this disease exists in a less annoying and less dangerous form for several years previous to the above aggravated symptoms characteristic of the loss of the teeth or life, I have thought best to tabulate its progress by treating it under four heads or divisions, as follows:

First. Where the margin of the gums shows decided inflammatory action, with some absorption of its substance, and bleeding at the slightest touch of the brush.

Second. Where the inflammation extends down over the thinner alveolar border, causing absorption of the bone as well as gum tissue, forming small pockets beneath the gum, filled with pus.

Third. Where the diseased action takes deeper hold, involving the thicker portions of the process, absorbing it most rapidly nearest the tooth, causing the tooth to sway back and forth for lack of most of its bony support.

Fourth and last stage Where the disease has swept away all the alveoli and much of the gum, the tooth being held in place by the conversion of the peridental membrane at the apex of the root into a tough ligamentous attachment.

The first and second stage is seldom noticed, only when it has reached the third stage does it challenge the attention of the practitioner; then to be treated by astringent washes or styptic tooth-powders, or by the more heroic treatment of slitting the gums between the teeth down to the bone with a thin thumb lancet, only to relieve the turgid gums temporarily by copious bleeding. The patient is informed that he has scurvy of the gums, or bone disease or old age disease, though the subject be not thirty years of age; or that the disease is hereiditary or constitutional; or that the patient is of a scrofulous diathesis.

So much of the old pathology and treatment. What saith the new? I plead a general denial of the old pathology and treatment, and offer the following point in proof of its falsity.

Present the worst case possible, one of those described in the first pages of this paper, and it shall make full and complete answer. Extract every tooth in said case and the patient will rapidly recover his health, his jaws and gums will immediately show an improved appearance; the gum tissue will change from a dark red and swollen condition to a light pink and healthy color, and the whole mouth in due time will present as healthy a base for an artificial denture as was ever seen. Now if it is a born disease, or heriditary, this sudden loss of the teeth would work no cure, but the disease should and would march straight forward until all the processes of both jaws were annihilated,. if so be, the patient lived so long. Not so however. The teeth being lost, the disease is arrested, active and suppurative inflammation and absorption of the parts subside into a curative and healthy action. New bone cells are deposited into the remaining alveoli until the apex or ridge of the jaws is rounded over and health reigns where a disgusting and wasting disease lately committed its ravages, and what is more and better, health, vigor and happiness light up the eye and crown the brow where sat inanition and gaunt despair.

This is no fancy picture, but a slight outline of the true portrait. I plead, secondly, an affirmation, and adduce the proof that the teeth themselves, with their accumulated accretions and roughened surfaces from whatever source derived, are the exciting cause of the disease under consideration. The teeth in perfect polish and cleanliness, at and under the margin of the gums. whether of animals or man, produce no inflamed action in that tissue. It can be artifically produced, however, by inserting a foreign body into or beneath its substance. If then diseased action can be set up by a foreign body, artificially introduced, it can be arrested and cured by withdrawing the same. And therefore, if the tooth becomes an extraneous body by reason of the accretions and the concretions upon it, near and under the free margin of the gum, inflammation ensues, as it certainly will, the true prophylactic and pathologic treatment surely would be to thoroughly and carefully remove said concretion, tartar and roughness—polish the tooth and let Nature take care of the rest. In two hour's time the inflamed and bleeding gum will assume a lighter color, its swollen tissue will begin to shrink to its normal thickness, will grow more tense and firm, and in twenty or thirty hours will grasp the neck of the tooth to the exclusion of all foreign matter.

I have thus pointed out the treatment of the disease in the first stage. The treatment of the second stage is the same, only be careful to reach the extreme limits of the diseased action. breaking up the diseased tissue and removing every particle of tartar from the tooth, and necrosed bone from the edge or margin of the process.

The third stage presents greater difficulties from the greater depth of the active line of disease, and demands a firm and skilled hand, a delicate and nice touch, and I might add, the transfer of the sense of sight to the finger ends.

The sense of touch must be so trained and cultivated that all foreign bodies can be readily distinguished from tooth-substance—live bone from necrosed bone, healthy from diseased tissue. This manipulation cannot be attained at once, but time and practice, with close and earnest study, will qualify and school the hand and embolden the true and sensitive mind to achieve success in the treatment of the third stage.

Of the treatment of the fourth and last stage little can be said, except that the loss of tooth or teeth is inevitable. The tooth is held in place by a cartilaginous attachment or condition of the investing root membrane, which holds it in place as a buoy is held in the water. The only alternative is extraction, for the alveoli is obliterated, and Nature refuses to reconstruct the socket new. The fiat has gone forth and we must acquiesce.

There is a point however, in the third stage where one or more teeth begin to grow loose, where restoration to health and firmness is not only possible, but quite certain, provided the operator is so self-possessed as to know when he has reached the utmost limit of the disease. If he fails to make a clean operation—that is, to go fully to the line of healthy bone, and to remove all foreign accumulation on the tooth—the disease is sure to show the fact *instanter*. If a speck of tartar not larger than a small grain of gunpowder be overlooked, the gum over it will manifest the fact by a reddened patch of the tissue several lines greater than the tartar underneath. We will assume, then, that operation is thoroughly performed. What then? Why this, surely: bone cells are deposited in the crater-like depression in which the tooth stands, and calcify around the tooth and make it firm and strong in position. The gum firmly grasps the root above this new-formed bone, and presents a perfectly healthy appearance.

Some mouths present all four stages of the disease at the same time, whilst others, the younger patients, present only the first or second. So far from being a disease of old age, cases are numerous at seventeen, and even fourteen years. These, however, are in the first stage, and blessed are the eyes of that man who recognizes it and applies the remedial treatment thus early. No medicinal treatment is needed, farther than an astringent wash, and myrrh tincture, full strength, touched to the gum with the end of the fingers, is best of all.—*Pennsylvania Journal of Dental Science.*

---

ARTICLE IV.

